# Supplementary material for: Machine learning-guided acyl-ACP reductase engineering for improved in vivo fatty alcohol production
Source: Nat Commun. 2021 Oct 5;12:5825. doi: 10.1038/s41467-021-25831-w (PMC8492656; doi:10.1038/s41467-021-25831-w)
Supplement: Supplementary file 1 — Supplemental Information [file 41467_2021_25831_MOESM1_ESM.docx]

**Machine learning-guided acyl-ACP reductase engineering for improved *in vivo* fatty alcohol production**

Greenhalgh *et al.*

**Supplementary Method 1. Gaussian process regression and UCB calculation**

During the training step of each round, we preprocessed the data and mean centered the encoding of the training set ($\mathbf{x}_{trn}$) the test set encoding ($\mathbf{x}_{tst}$), and the activity values for the training set ($\mathbf{y}_{trn}$). Then, to train the model, we follow Algorithm 2.1 presented in Rasmussen and Williams^1^. First, we evaluate the kernel function between all pairs of training sequences ($K$) and between the training sequences and test sequences ($\mathbf{k}_{*}$):

$K=\sigma^{2}\mathbf{x}_{trn}\cdot\mathbf{x}_{trn}^{T}$ (1)

$\mathbf{k}_{*}=\sigma^{2}\mathbf{x}_{trn}\cdot\mathbf{x}_{tst}^{T}$ (2)

where $\sigma^{2}$ is the hyperparameter used to regularize the model. Then, we perform a Cholesky decomposition:

$L=\mathrm{Cholesky}\left( K+\sigma_{n}^{2}I \right)$ (3)

where $\sigma_{n}^{2}$ is the noise level (and in this case was set equal to 1) and $I$ is the identity matrix. $L$ is a lower triangular matrix that can be used to efficiently solve the following:

$\boldsymbol{\alpha}=L^{T}(L\backslash\mathbf{y}_{trn})$ (4)

The predicted mean ($\mathbf{y}^{*}$) can then be calculated as:

$\mathbf{y}^{*}=\mathbf{k}_{*}^{T}\boldsymbol{\alpha}+Y_{mean}$ (5)

where $Y_{mean}$ is the mean activity value of the training sequences. The confidence intervals ($CI$) are then determined by calculating the variance ($\mathbf{v}^{*}$**)** as follows:

$\mathbf{v}=L\backslash\mathbf{k}_{*}$ (6)

$\mathbf{v}^{*}=diag\left( \sigma^{2}\mathbf{x}_{tst}\cdot\mathbf{x}_{tst}^{T} \right)-\mathbf{v}^{T}\mathbf{v}$ (7)

$CI=\sqrt{\mathbf{v}^{*}}$ (8)

Finally, the UCB can be calculated by summing $\mathbf{y}^{*}$ and $CI$ for each sequence.

**Supplementary Table 1. Strain list.**

| **Strain** | **Description** | **Source/Reference** |
| --- | --- | --- |
| *Escherichia coli* DH5α | n/a | Lucigen |
| *Escherichia coli* BL21 DE3 | n/a | Lucigen |
| *Escherichia coli* 10 G Supreme | n/a | Lucigen |
| *E. coli* RL08ara | *E. coli* K-12 MG1655 ΔfadD ΔaraBAD ΔaraFGH Φ(ΔaraEp P_CP18_-araE) | Lennen *et al*.^2^ |
| *E. coli* CM24 | *E. coli* LS5218 ΔfadE ΔatoC ΔldhA ΔackApta ΔadhE ΔpoxB ΔfrdABCD ΔydiO ΔfadBA ΔfadIJ ΔfadD | Mehrer *et al*.^3^ |

**Supplementary Table 2. Key plasmids.**

| **Name** | **Description** | **Source** |
| --- | --- | --- |
| pET 28 MA-ACR | ACR from *Marinobacter aqueolei* VT8 fused to maltose binding protein (MBP) on pET 28 backbone, T7 promoter KmR | Rung Yi Lai, (currently Suranaree University of Technology), while a post-doc at UW-Madison |
| pBTRKtrc | Ptrc promoter, pBBR1 origin, KanR | Youngquist *et al*.^4^ |
| pBTRCK MA-ACR | ACR from *Marinobacter aqueolei* VT8 fused to maltose binding protein (MBP) on pBTRCKtrc backbone | Youngquist *et al*.^4^ |
| pBTRCK MB-ACR | ACR from *Marinobacter BSs20148* fused to maltose binding protein (MBP) on pBTRCKtrc backbone | Mehrer *et al*.^3^ |
| pBTRCK MT-ACR | ACR from *Methylibium Sp. T29* fused to maltose binding protein (MBP) on pBTRCKtrc backbone | Mehrer *et al*.^3^ |
| pACYC-seFadBA | FadBA from *Salmonella enterica*, pACYC origin, Trc promoter, CmR | Mehrer *et al*.^3^ |
| pTRC99A-vhTER-fdh | Trans-enyol-CoA reductase (TER) from *Vibrio harveyi* and formate dehydrogenase from *Candida boindinii*, pBR322 origin, Trc Promoter, AmpR | Mehrer *et al*.^3^ |
| pET 28 Ec-ACP | Apo-Acyl Carrier Protein (ACP) from *E. coli* | Hernández-Lozada *et al*.^5^ |
| pET 28 Vh-AasS | AasS from *V. harveyi* | Hernández-Lozada *et al*.^5^ |
| pET 28 Bs-Sfp | Sfp from Bascillus subtilis | Hernández-Lozada *et al*.^5^ |
| pLacIRARE rTEV (pQE60) | TEV Protease | Hernández-Lozada *et al*.^5^  (originally from Hazel Holden Lab) |

**Supplementary Table 3. Protein structure templates used for homology models.**

| **Template (PDB)** | **Protein function** | **%ID to MA-ACR** |
| --- | --- | --- |
| [3m1a.A](https://www.rcsb.org/structure/3M1A) | Short-chain dehydrogenase | 40 |
| [3rkr.A](https://www.rcsb.org/structure/3RKR) | Short-chain oxidoreductase | 37 |
| [3rih.A](https://www.rcsb.org/structure/3RIH) | Putative short-chain dehydrogenase or reductase | 36 |
| [3afm.B](https://www.rcsb.org/structure/3AFM) | Aldose reductase | 36 |
| [3afn.B](https://www.rcsb.org/structure/3AFN) | Aldose reductase | 36 |
| [4bmv.A](https://www.rcsb.org/structure/4BMV) | Short-chain dehydrogenase | 36 |

**Supplementary Table 4. Details of UCB optimization rounds.**

|  | **Data used for model training** | **Model used for design** | **Sequence design criteria** | **# of sequences designed** | **# of sequences tested** | **Allowed block subs** | **Classifier activity threshold (mg/L)** | **# of sequences predicted to be active** | **GNB AUC** | **Method for** $\boldsymbol{\sigma}^{\boldsymbol{2}}$  **selection** | $\boldsymbol{\sigma}^{\boldsymbol{2}}$ | **GP model correlation coefficient** | **Sum of squared error** |
| --- | --- | --- | --- | --- | --- | --- | --- | --- | --- | --- | --- | --- | --- |
| Initialization | None | None | Max MI | 20 | 20 | 5 | N/A | N/A | N/A | N/A | N/A | N/A | N/A |
|  |  |  |  |  |  |  |  |  |  |  |  |  |  |
| UCB1* | CM24 + BL21 | GPR with Hamming kernel | UCB | 10 | 5 | 5 | N/A | N/A | None | Inspection | 0.01 | Not calculated | Not calculated |
| UCB2** | RL08ara | NB, GPR with Hamming kernel | UCB positive | 10 | 7 | 5 | 1.1 | 1268 | Not calculated | Inspection | 0.433 | 0.192 | 11.46 |
| UCB3 | RL08ara | NB, GPR with Hamming kernel | UCB positive | 10 | 7 | 5 | 1.1 | 1414 | Not calculated | Inspection | 0.433 | -0.368 | 38.77 |
| UCB4 | RL08ara | NB, GPR with Hamming kernel | UCB positive | 10 | 5 | 5 | 1.1 | 1341 | 0.7086 | Maximize correlation coefficient | 0.00658 | -0.275 | 23.91 |
| UCB5 | RL08ara | NB, GPR with structure kernel | UCB positive | 10 | 4 | 2 | 1.1 | 1951 | 0.7472 | Minimize sum of squared error | 0.000167 | 0.189 | 19.2 |
| UCB6*** | RL08ara | NB, GPR with structure kernel | UCB positive | 10 | 9 | 2 | 4 | 715 | 0.7886 | Minimize sum of squared error | 0.0599 | 0.632 | 5.2 |
| UCB7 | RL08ara | NB, GPR with structure kernel | UCB positive | 10 | 10 | 2 | 4 | 714 | 0.81 | Maximize correlation coefficient | 0.0359 | 0.384 | 11.17 |
| UCB8 | RL08ara | NB, GPR with structure kernel | UCB positive | 10 | 7 | 2 | 4 | 587 | 0.869 | Inspection | 0.0774 | 0.472 | 13.06 |
| UCB9 | RL08ara | NB, GPR with structure kernel | UCB positive | 10 | 10 | 5 | 4 | 614 | 0.8799 | Maximize correlation coefficient | 0.00774 | 0.466 | 25 |
| UCB10 | RL08ara | NB, GPR with structure kernel | UCB positive | 12 | 9 | 5 | 8 | 323 | 0.9757 | Minimize sum of squared error | 0.0215 | 0.786 | 5.47 |
|  | | | | | | | | | | | | | |
| *Cross validation was not used for hyperparameter tuning during the first round | | | | | | | | | | | | | |
| **Sequences in this round were non-UCB-optimal due to a coding error, but the data generated from these sequences was still valuable for training models. | | | | | | | | | | | | | |
| ***The labels for two sequences in this round were accidentally swapped during model training | | | | | | | | | | | | | |

**Supplementary Table 5. Fatty alcohol titers of chimeric ATRs characterized in RL08ara both during the UCB phase and final validation.**

|  | **Block_seq** | **UCB titer (mg/L)** | **n (UCB)** | **Finalized titer (mg/L)** | **n (finalized)** |
| --- | --- | --- | --- | --- | --- |
| **ATR-01** | A-ATAATTBB | 1.5 ± 0.3 | 3 | 1.5 ± 0.3 | 3 |
| **ATR-02** | A-TATTTTAB | 0.8 | 1 | 0.8 | 1 |
| **ATR-03** | A-TTTTBTBA | 0.9 | 1 | 0.9 | 1 |
| **ATR-04** | A-ATTBAATB | 0.6 | 1 | 0.6 | 1 |
| **ATR-05** | A-ABTATTTA | 0.9 | 1 | 0.9 | 1 |
| **ATR-06** | A-TAATBTTB | 0.9 | 1 | 0.9 | 1 |
| **ATR-07** | A-AATAATBT | 0.8 | 1 | 0.8 | 1 |
| **ATR-08** | A-ATTABTAT | 0.8 | 1 | 0.8 | 1 |
| **ATR-09** | A-TBATATAB | 1 | 1 | 1 | 1 |
| **ATR-10** | A-TTBAATTA | 2.8 ± 0.1 | 3 | 2.8 ± 0.1 | 3 |
| **ATR-11** | A-ATBTATTA | 0.7 | 1 | 0.7 | 1 |
| **ATR-12** | A-BAABTTAA | 0.9 | 1 | 0.9 | 1 |
| **ATR-13** | A-BTTAAATB | 0.8 | 1 | 0.8 | 1 |
| **ATR-14** | A-TBBTTABT | 1 | 1 | 1 | 1 |
| **ATR-15** | A-TBABATTT | 0.7 | 1 | 0.7 | 1 |
| **ATR-16** | A-BBATBATT | 0.7 | 1 | 0.7 | 1 |
| **ATR-17** | A-BBTTATBA | 0.7 | 1 | 0.7 | 1 |
| **ATR-18** | A-TTABTABA | 2.2 ± 0.2 | 3 | 2.2 ± 0.2 | 3 |
| **ATR-19** | A-TBTABAAA | 0.8 | 1 | 0.8 | 1 |
| **ATR-20** | A-BTBATTAT | 2.6 ± 0.2 | 3 | 2.6 ± 0.2 | 3 |
| **ATR-21** | A-AABTTAAT | 0.8 | 1 | 0.8 | 1 |
| **ATR-22** | A-BABBTATA | 1 | 1 | 1 | 1 |
| **ATR-23** | A-ATBBBAAT | 3.8 ± 0.3 | 3 | 3.8 ± 0.3 | 3 |
| **ATR-24** | A-TABATABT | 0.8 | 1 | 0.8 | 1 |
| **ATR-25** | A-BTBTTAAB | 5 ± 0.9 | 3 | 5 ± 0.9 | 3 |
| **ATR-26** | A-TAAABTBB | 3.6 ± 0.6 | 3 | 3.6 ± 0.6 | 3 |
| **ATR-27** | A-TTBTTTAA | 4 ± 1 | 3 | 4 ± 1 | 3 |
| **ATR-28** | A-TBAAATAA | 2.7 ± 0.2 | 3 | 2.7 ± 0.2 | 3 |
| **ATR-29** | A-TBTTATTB | 0.5 ± 0.1 | 3 | 0.5 ± 0.1 | 3 |
| **ATR-30** | A-TAATAATA | 0.6 ± 0.1 | 3 | 0.6 ± 0.1 | 3 |
| **ATR-31** | A-TBTTTATB | 0.6 ± 0.2 | 3 | 0.6 ± 0.2 | 3 |
| **ATR-32** | A-TBTATATA | 0.7 ± 0.2 | 3 | 0.7 ± 0.2 | 3 |
| **ATR-33** | A-BTTAATBA | 0.9 ± 0.2 | 3 | 1 ± 0.2 | 5 |
| **ATR-34** | A-BTATBTTA | 4 ± 0.1 | 3 | 5.2 ± 1.6 | 5 |
| **ATR-35** | A-ABBATABA | 0.8 ± 0.1 | 3 | 1 ± 0.3 | 5 |
| **ATR-36** | A-AABBTTBA | 1 ± 0.1 | 3 | 1.3 ± 0.4 | 5 |
| **ATR-37** | A-BABTTTBA | 1.2 ± 0.2 | 3 | 1.4 ± 0.2 | 5 |
| **ATR-38** | A-AABTTTBA | 1.2 ± 0.1 | 3 | 1.4 ± 0.3 | 5 |
| **ATR-39** | A-AABATABA | 1 ± 0.2 | 3 | 1.1 ± 0.3 | 5 |
| **ATR-40** | A-BTBBBABB | 21 ± 2 | 3 | 21 ± 2 | 3 |
| **ATR-41** | A-ATAAAAAB | 27 ± 7 | 3 | 27 ± 7 | 3 |
| **ATR-42** | A-AABABAAA | 12 ± 1 | 3 | 12 ± 1 | 3 |
| **ATR-43** | A-TTBTBTTT | 2.9 ± 0.3 | 3 | 2.9 ± 0.3 | 3 |
| **ATR-44** | A-AAABBAAA | 12.5 ± 1 | 2 | 12.5 ± 1 | 2 |
| **ATR-45** | A-AAABAAAB | 33 ± 6 | 3 | 33 ± 6 | 3 |
| **ATR-46** | A-AABAAAAB | 19 ± 2 | 3 | 19 ± 2 | 3 |
| **ATR-47** | A-BBBTBABB | 8.1 ± 0.8 | 3 | 8.1 ± 0.8 | 3 |
| **ATR-48** | A-BAAAAAAB | 15.8 ± 2 | 3 | 15.8 ± 2 | 3 |
| **ATR-49** | A-ABAAAAAT | 3.6 ± 0.1 | 2 | 3.6 ± 0.1 | 2 |
| **ATR-50** | A-BBBBBATB | 1.4 ± 0.1 | 2 | 1.4 ± 0.1 | 2 |
| **ATR-51** | A-BBBBBTAB | 20 ± 3 | 2 | 20 ± 3 | 2 |
| **ATR-52** | A-ABBAAAAA | 12 ± 3 | 2 | 12 ± 3 | 2 |
| **ATR-53** | A-ABBBBTBB | 21 ± 1 | 2 | 21 ± 1 | 2 |
| **ATR-54** | A-AABAAAAT | 4.4 ± 0.1 | 2 | 4.4 ± 0.1 | 2 |
| **ATR-55** | A-BBBBBTTB | 1.3 ± 0.2 | 2 | 1.3 ± 0.2 | 2 |
| **ATR-56** | A-ATBAAAAA | 14 ± 4 | 2 | 14 ± 4 | 2 |
| **ATR-57** | A-BBBABTBB | 9.5 ± 0.6 | 2 | 9.5 ± 0.6 | 2 |
| **ATR-58** | A-AAAABATA | 0.8 ± 0.1 | 3 | 0.8 ± 0.1 | 3 |
| **ATR-59** | A-BBBBBAAB | 49.1 ± 0.9 | 4 | 49.1 ± 0.9 | 4 |
| **ATR-60** | A-ATABAAAA | 19 ± 1 | 2 | 19 ± 1 | 2 |
| **ATR-61** | A-AABBAAAA | 12 ± 2 | 3 | 12 ± 2 | 3 |
| **ATR-62** | A-BABBBABB | 37 ± 5 | 2 | 37 ± 5 | 2 |
| **ATR-63** | A-ABBBBABB | 36 ± 4 | 3 | 36 ± 4 | 3 |
| **ATR-64** | A-AAAABAAB | 13.2 ± 0.6 | 2 | 13.2 ± 0.6 | 2 |
| **ATR-65** | A-ABAAAAAB | 14 ± 2 | 2 | 14 ± 2 | 2 |
| **ATR-66** | A-BTBBBTBB | 18.8 ± 0.7 | 2 | 18.8 ± 0.7 | 2 |
| **ATR-67** | A-BBBABABB | 7 ± 5 | 2 | 7 ± 5 | 2 |
| **ATR-68** | A-BTBBBATB | 7.7 ± 0.4 | 2 | 7.7 ± 0.4 | 2 |
| **ATR-69** | A-BTBBBAAB | 54 ± 6 | 4 | 54 ± 6 | 4 |
| **ATR-70** | A-BBBBAAAB | 39 ± 4 | 2 | 39 ± 4 | 2 |
| **ATR-71** | A-ABBBBAAB | 50 ± 4 | 2 | 45 ± 14 | 4 |
| **ATR-72** | A-BABBBAAB | 34 ± 6 | 2 | 34 ± 6 | 2 |
| **ATR-73** | A-BBABBATB | 2.8 ± 0.4 | 2 | 2.8 ± 0.4 | 2 |
| **ATR-74** | A-ATBBBABB | 19 ± 2 | 2 | 19 ± 2 | 2 |
| **ATR-75** | A-ABBBBATB | 2.4 ± 0.3 | 2 | 2.4 ± 0.3 | 2 |
| **ATR-76** | A-BABBBATB | 4.3 ± 0 | 2 | 4.3 ± 0 | 2 |
| **ATR-77** | A-AABBBABB | 35.5 ± 0 | 2 | 35.5 ± 0 | 2 |
| **ATR-78** | A-ATBBATAB | 41 ± 10 | 2 | 41 ± 10 | 2 |
| **ATR-79** | A-BTABBAAB | 54 ± 1 | 2 | 54 ± 1 | 2 |
| **ATR-80** | A-BTBBAAAB | 60 ± 4 | 3 | 60 ± 4 | 3 |
| **ATR-81** | A-ATBTBAAB | 24 ± 24 | 4 | 24 ± 24 | 4 |
| **ATR-82** | A-BTABAAAB | 54 ± 3 | 2 | 42 ± 21 | 3 |
| **ATR-83** | A-ATBBAAAB | 61 ± 6 | 4 | 54 ± 11 | 11 |
| **ATR-84** | A-AABBBAAB | 36 ± 10 | 2 | 36 ± 10 | 2 |
| **ATR-85** | A-ATABBAAB | 48 ± 7 | 2 | 48 ± 7 | 2 |
| **ATR-86** | A-ATBBBAAB | 56 ± 4 | 2 | 45 ± 19 | 3 |
| **ATR-87** | A-ATABAAAB | 50 ± 12 | 2 | 50 ± 12 | 2 |
| **ATR-88** | A-ATBBBTAB | 33 ± 3 | 2 | 33 ± 3 | 2 |
| **ATR-89** | A-AABBAAAB | 40 ± 4 | 2 | 40 ± 4 | 2 |
| **ATR-90** | A-BTBAAAAB | 37 ± 2 | 2 | 37 ± 2 | 2 |
| **ATR-91** | A-ATBABAAB | 16 ± 3 | 2 | 16 ± 3 | 2 |
| **ATR-92** | A-ABBBAAAB | 35 ± 4 | 2 | 35 ± 4 | 2 |
| **ATR-93** | A-BTBBATAB | 35 | 1 | 35 | 1 |
| **Fusion B-A** | B-AAAAAAAA | N/A | N/A | 10.6 ± 3 | 4 |
| **Fusion B-T** | B-TTTTTTTT | N/A | N/A | 6 ± 2 | 4 |
| **Fusion T-A** | T-AAAAAAAA | N/A | N/A | 1 ± 0 | 4 |
| **Fusion T-B** | T-BBBBBBBB | N/A | N/A | 2 ± 0 | 4 |
| **MA-ACR (Parent A)** | A-AAAAAAAA | 12 ± 3 | 13 | 11 ± 3 | 26 |
| **MB-ACR** | B-BBBBBBBB | 23 ± 1 | 3 | 26 ± 8 | 7 |
| **MT-ACR*** | T-tTTTTTT | 5 ± 1 | 3 | 4 ± 1 | 7 |
| **Parent B (Fusion A-B)** | A-BBBBBBBB | 39 ± 5 | 5 | 37 ± 8 | 13 |
| **Parent T (Fusion A-T)** | A-TTTTTTTT | 8 ± 1 | 3 | 6 ± 2 | 10 |

The average titer is represented as the mean ± SD. The number of replicates (n) indicates the number of cultures derived from individual colonies tested. Titers reported in ‘Finalized Titer’ column of Supplementary Table 5 are the final titers averaged over all experimental replicates, including additional validation experiments. For this reason, some of the final titers reported in Supplementary Table 5 differ slightly from the titers shown in the Figure 2 of the main text, which reflect average titers based on fewer replicates during the UCB optimization process (the titers presented in Figure 2 are shown in the ‘UCB Titer’ column of Supplementary Table 5). Specifically, the titer of ATR-83 was originally 61 mg/L (n = 4), making it the top enzyme sequence during the UCB optimization. This is why ATR-83 was chosen for further characterization. Additional *in vivo* experiments performed on ATR-83 caused its average to drift down to 54 mg/L (n = 11) and below the next highest enzyme’s (ATR-80) titer of 60 mg/L (n = 3). We performed a Welch’s T-test and found the average titers of ATR-80 and ATR-83 are not statistically significant (*p*=0.18). The WT MT-ACR sequence in the first block (1t) of the ATR domain differs from the sequence of the block used to make chimeric sequences (1T) containing that block by a one amino acid swap (N to H).

**Supplementary Table 6. Amino-acid sequences of blocks and sequence elements.**

|  | **Element/block** | **Amino acid sequence** |
| --- | --- | --- |
| **MBP Tag** | **Maltose binding protein (MBP) tag** | MKIEEGKLVIWINGDKGYNGLAEVGKKFEKDTGIKVTVEHPDKLEEKFPQVAATGDGPDIIFWAHDRFGGYAQSGLLAEITPDKAFQDKLYPFTWDAVRYNGKLIAYPIAVEALSLIYNKDLLPNPPKTWEEIPALDKELKAKGKSALMFNLQEPYFTWPLIAADGGYAFKYENGKYDIKDVGVDNAGAKAGLTFLVDLIKNKHMNADTDYSIAEAAFNKGETAMTINGPWAWSNIDTSKVNYGVTVLPTFKGQPSKPFVGVLSAGINAASPNKELAKEFLENYLLTDEGLEAVNKDKPLGAVALKSYEEELVKDPRIAATMENAQKGEIMPNIPQMSAFWYAVRTAVINAASGRQTVDEALKDAQTNSSSNNNNNNNNNNLGIEGRISEF |
| **AHR Domains** | **AHR domain -A** | NYFLTGGTGFIGRFLVEKLLARGGTVYVLVREQSQDKLERLRERWGADDKQVKAVIGDLTSKNLGIDAKTLKSLKGNIDHVFHLAAVYDMGADEEAQAATNIEGTRAAVQAAEAMGAKHFHHVSSIAAAGLFKGIFREDMFEEAEKLDHPYLRTKHESEKVVREECKVPFRIYRPGMVIGHSETGEMDKVDGPYYFFKMIQKIRHALPQWVPTIGIEGGRLNIVPVDFVVDALDHIAHLEGEDGNCFHLVDSDPYKVGEILNIFCEAGHAPRMGMRIDSRMFGFIPPFIRQSIKNLPPVKRITGALLDDMGIPPSVMSFINYPTRFDTRELERVLKGTDIEVPRLPSYAPVIWDY |
|  | **AHR domain B** | NYFVTGGTGFIGRFLIAKLLARGAIVHVLVREQSVQKLADLREKLGADEKQIKAVVGDLTAPSLGLDKKTLKQLSGKIDHFFHLAAIYDMSASEESQQAANIDGTRAAVAAAEALEAGIFHHVSSIAVAGLFKGTFREDMFAEAGKLDHPYFRTKHESERVVRDDCKVPFRIYRPGLVIGDSATGDMDKVDGPYYFFKMIQKIRGALPQWVPTIGIEGGRLNIVPVNFVADALDHIAHLPNEDGKCFHLVDSDPYKVGEILNIFCEAGHAPKMGMRIDSRMFGFVPPFIRQSLKNLPPVKRMGRALLDDLGIPASVLSFINYPTRFDARETERVLQGTGIEVPRLPDYAPVIWDY |
|  | **AHR domain T** | QYFVTGATGFIGKRLVRKLLDRRGSTVHFLLRPESERKLPELLAYWGLSGAAKARAVPVYGDLTAKKLGVAADAIKALKGRIDAIYHLAAVYDLGADEAAQVQVNIEGTRSAVEFAQAIQAGHFHHVSSIAAAGLYEGVFREDMFDEAEGLDHPYFMTKHESEKIVRKECKLPWTVFRPAMVVGDSTTGEMDKIDGPYYFFKLIQRMRQLLPPWMPAVGLEGGRVNIVPVDFVVAALDHISHAKLELDRRCFHLVDPVGYRVGDVLDIFGKAAHAPKMNLFVNAALLGFIPKSVKKGLMALAPVRRIRNAVMKDLGLPEDMLTFVNYPTRFDCRDTQAALKGSGIECPNLKDYAWRLWDY |
| **ATR Domain Blocks** | **1A** | WERNLDPDLFKDRTLKGTVEGKVCV |
|  | **1B** | WERNLDPDLFKDRTLRGTVEGKVCV |
|  | **1T** | WERNLDPDLFIDRSLRGTVGGKVVL |
|  | **1t*** | WERHLDPDLFIDRSLRGTVGGKVVL |
|  | **2A** | VTGATSGIGLATAEKLAEAGAILVIGARTKETLDEVAASLEAKGGNVHAYQCDFS |
|  | **2B** | VTGATSGIGLATAEKLADAGAILVIGARTQETLDQVSAQLNARGADVHAYQCDFA |
|  | **2T** | VTGGSSGIGLAAACKFAEAGAVTVICARDADKLDEAVKEIKAFAGKEARVFSYSVDIA |
|  | **3A** | DMDDCDRFVKTVLDNHGHVDVLVNN |
|  | **3B** | DMDACDRFIQTVSENHGAVDVLINN |
|  | **3T** | DEAGCKAFLEALQAEHGGVDFLINN |
|  | **4A** | AGRSIRRSLALSFDRFHDFERTMQLNY |
|  | **4B** | AGRSIRRSLDKSFDRFHDFERTMQLNY |
|  | **4T** | AGRSIRRAIENSYERFHDFERTMQLNY |
|  | **5A** | FGSVRLIMGFAPAMLERRRGHVVNISSIGVLTNAPRFSAYVSSKSALDAFSRCAAAEWSDRNVTF |
|  | **5B** | FGSLRLIMGFAPAMLERRRGHIINISSIGVLTNSPRFSAYVASKSALDSFSRCAAAEWSDRRVCF |
|  | **5T** | FGCLRVTMGVLPGMVAKRKGHVVNISSIGVLTNAPRFSAYVASKAALDAWTRCASSEYADTGISF |
|  | **6A** | TTINMPLVKTPMIAPTKIYDSVPT |
|  | **6B** | TTINMPLVKTPMIAPTKIYDSVPT |
|  | **6T** | TTINMPLVRTPMIAPTKIYNNVPT |
|  | **7A** | LTPDEAAQMVADAIVYRPKRIATRLGVFAQVLHALAPKMGEIIMNTGYRM |
|  | **7B** | LSPEEAADMVVNAIVYRPKRIATRMGVFAQVLNAVAPKASEILMNTGYKM |
|  | **7T** | LAPEEAADMIAQACVYKPVRIATRLGTAGQVLHALAPRVAQIVMNTSFRM |
|  | **8A** | FPDSPAAAGSKSGEKPKVSTEQVAFAAIMRGIYW* |
|  | **8B** | FPDSMPKKGKEVSAEKGASTDQVAFAAIMRGIHW* |
|  | **8T** | FPDSEAAKGEKGAKPQLSAEAVALQQMMRGIHF* |

**Supplementary Table 7. Primer list.**

| **Name** | **Sequence** | **Purpose** |
| --- | --- | --- |
| rJCG5 | CTGGACCCGGACCTGTTCATCGATCGTAGCTTGCGT | Clone MT-ACR (domain 2) onto domain 1 of MA-ACR and backbone via Gibson assembly |
| rJCG6 | GAACAGGTCCGGGTCCAG | Amplify MA-ACR domain 1 and pET 22 vector backbone for Gibson assembly of MT- and MB-ACR fusions |
| rJCG7 | TTTGTTAGCAGCCGGATCTTAAAAATGAATACCGCG | Clone MT-ACR (domain 2) onto domain 1 of MA-ACR and backbone via Gibson assembly |
| rJCG8 | CTGGACCCGGACCTGTTCAAAGATCGCACTCTCAGA | Clone MB-ACR (domain 2) onto domain 1 of MA-ACR and backbone via Gibson assembly |
| rJCG9 | TTTGTTAGCAGCCGGATCTTACCAGTGGATACCACG | Clone MB-ACR (domain 2) onto domain 1 of MA-ACR and backbone via Gibson assembly |
| rJCG10 | GATCCGGCTGCTAACAAA | Amplify MA-ACR domain 1 and pET 22 vector backbone for Gibson assembly of MB-- and MT-ACR fusions |
| rJCG11 | GGCTATAACGGGCTCGCTGAAG | Remove BsaI site from MBP |
| rJCG12 | TTTATCGCCGTTAATCCAGAT | Remove BsaI site from MBP |
| rJCG13 | GGTCTCGGTAGTCCCAGATAACCGG | Put BsaI site into MA-ACR domain 1 |
| rJCG14 | GGTCTCGTAAGATCCGGCTGCTAA | Put BsaI site onto pET 28 after MA-ACR domain 2 |
| rJCG15 | CCGGATGCTCAACGG | For verifying sequence of MBP w/o BsaI site |
| rJCG16 | GTGAAATCATGCCGAACATC | Sequencing MBP-ACR Junction |
| rJCG17 | AATTACCCGACCCGT | For sequencing ATR domain |
| rJCG18 | CCCATCGGTCTCGTAAGATCCGGCTGCTAA | For amplifying pET 28/ ATR 1 backbone with extra bases after the BsaI sites |
| rJCG19 | GCCTGAGGTCTCGGTAGTCCCAGATAACCGG | For amplifying pET 28/ ATR 1 backbone with extra bases after the BsaI sites |
| rJCG29 | TGTAAAACGACGGCCAGT | Amplifying inserts from Twist plasmids containing ATR blocks. Similar to M13 Forward (-20) sequencing primer |
| rJCG30 | CACACAGGAAACAGCTATGAC | Amplifying inserts from Twist plasmids containing ATR blocks. Similar to M13 Reverse (-27) sequencing primer |
| rJCG36 | GGC TAT AAC GGG CTC GC | Fixing deletion in MBP |
| rJCG37 | TTT ATC GCC GTT AAT CCA GAT T | Fixing deletion in MBP |
| rJCG38 | CATACTTGAACGCATAAC | Sequencing MBP |
| rJCG39 | GGTATATCTCCTTCTTAAAGTTA | Making empty pET 28 vector |
| rJCG40 | TAAGATCCGGCTGCTAAC | Making empty pET 28 vector |
| rJCG41 | CCCCACACTACCATCGG | Eliminating BsaI site in 5S RNA terminator region of pBTRCk plasmid |
| rJCG42 | GCTCCCCATGCGAGAGTAG | Eliminating BsaI site in 5S RNA terminator region of pBTRCk plasmid |
| rJCG43 | GCTCGCTGAAGTCGGTAAGAA | Eliminating BsaI site in MBP in MA-ACR on the pBTRCk plasmid |
| rJCG44 | CCGTTATAGCCTTTATCGCC | Eliminating BsaI site in MBP in MA-ACR on the pBTRCk plasmid |
| rJCG45 | AAGACAGGTCTCGGTAGTCCCAGATAACCGGG | Putting Golden-Gate sites in MA-ACR on the pBTRCk plasmid |
| rJCG46 | GTCCGAGGTCTCGTAAGTAGACCATCATCACCATCATCA | Putting Golden-Gate sites in MA-ACR on the pBTRCk plasmid |
| rJCG47 | TCTGGTCTCGCTACTGGGAGCGCAATCT | Put Golden-Gate sites back on chimeric ATR sequence for transferring from pET 28 to pBTRCk |
| rJCG48 | CGAGGTCTCGCTTACCAGTATATCCCCCGC | Put Golden-Gate sites back on chimeric ATR sequence for transferring from pET 28 to pBTRCk |
| rJCG49 | CGAGGTCTCGCTTACCAGTGGATACCACGC | Put Golden-Gate sites back on chimeric ATR sequence for transferring from pET 28 to pBTRCk |
| rJCG50 | CGAGGTCTCGCTTAAAAATGAATACCGCGCATC | Put Golden-Gate sites back on chimeric ATR sequence for transferring from pET 28 to pBTRCk |
| rJCG51 | ATGCTATGGTCCTTGTTGGT | Make empty pBTRCK plasmid |
| rJCG52 | TAAGCTGTTTTGGCGGATG | Make empty pBTRCK plasmid |
| rJCG53 | TGGCAGGGTCTCGTCTGAAATCCTTCCCTCGATC | Make new Golden-Gate backbone for rebuilding parent enzymes |
| rJCG54 | ATACTAGGTCTCGCAGAATTCCAATATTTCGTTACCG | Re-clone WT MT-ACR to get rid of His Tag |
| rJCG55 | ATACTAGGTCTCGCAGAATTCAATTATTTTGTGACCG | Re-clone WT MB-ACR to get rid of His Tag |
| rJCG56 | GTAAAGGGTCTCGTGCGTTATTCACCAGTACATCC | Fix block 3 parent A (MA-ACR) overhangs |
| rJCG57 | GTAAAGGGTCTCGTGCGTTGTTAATCAGTACGTCC | Fix block 3 parent B overhangs |
| rJCG58 | GTAAAGGGTCTCGTGCGTTGTTGATTAAGAAATCTACTC | Fix block 3 parent T overhangs |
| rJCG59 | CCTACAGGTCTCGCGCAGGTCGCTCCATCCGC | Fix block 4 parent A (MA-ACR) overhangs |
| rJCG60 | CCTACAGGTCTCGCGCAGGTCGCAGCATCCGT | Fix block 4 parent B overhangs |
| rJCG61 | CCTACAGGTCTCGCGCAGGACGTAGCATCCGTCG | Fix block 4 parent T overhangs |
| rJCG62 | ATTGTGCCGGTGGATTTC | Sequencing ACR Golden-Gate junction |
| rJCG63 | GTAAGGAATGTAAGCTGCCATG | Sequencing MT-ACR (internal) |
| rJCG64 | AAGACAGGTCTCGGTAGTCCCAGATAACGGGGG | Cloning MB-ACRs AHR into a backbone vector |
| rJCG65 | AAGACAGGTCTCGGTAGTCCCATAAGCGCCACG | Cloning MT-ACRs AHR into a backbone vector |
| rJCG66 | GTCGAAGGTCTCGCTA CTG GGA GCG CAA TCT G | Cloning ACR chimeras from pBTRCk vector back into pET 28 vector, AHR side |
| rJCG67 | GTCGAAGGTCTCGCTT ACC AGT ATA TCC CCC GCA TAA TCG | Cloning ATR chimeras from pBTRCK vector back into pET 28 vector, ATR side parent 1 |
| rJCG68 | GTCGAAGGTCTCGCTT ACC AGT GGA TAC CAC GC | Cloning ATR chimeras from pBTRCK vector back into pET 28 vector, ATR side parent 2 |
| rJCG69 | GTCGAAGGTCTCGCTT AAA AAT GAA TAC CGC GCA TC | Cloning ATR chimeras from pBTRCK vector back into pET 28 vector, ATR side parent 3 |
| rJCG70 | TAATACGACTCACTATAGGG | T7 Promoter (sequencing) |
| rJCG71 | GCTAGTTATTGCTCAGCGG | T7 Terminator (sequencing) |
| rJCG72 | CTACAGGGCGCGTCCCATTCGC | pET50downstream_rev (sequencing) |
| rJCG73 | GTCGAAGGTCTCGTAAGTGCTGTTTTGGCGGATGA | Amplifying MB-ACR backbone to make constructs with MB-ACRs AHR domain in pBTRCK |
| rJCG74 | GTCGAAGGTCTCGGTAGTCCCAGATAACGGGGGC | Amplifying MB-ACR backbone to make constructs with MB-ACRs AHR domain in pBTRCK |
| rJCG75 | GTCGAAGGTCTCGCAGAATTCAATTATTTTGTGACCGG | Amplifying MB-ACR Aldehyde reductase domain along with rJCG74 in pBTRCK |
| rJCG76 | GTCGAAGGTCTCGGTAGTCCCATAAGCGCCACG | Amplifying MT-ACR aldehyde reductase domain along with rJCG 77 in pBTRCK |
| rJCG77 | GTCGAAGGTCTCGCAGAATTCCAATATTTCGTTACCG | Amplifying MT-ACR aldehyde reductase domain along with rJCG 76 in pBTRCK |


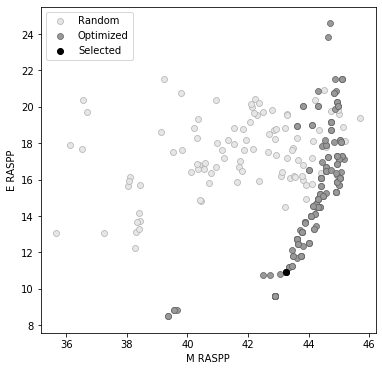


**Supplementary Figure 1**. **RASPP chimeric enzyme library design.** The SCHEMA-RASPP algorithm was used to identify sets of breakpoints that simultaneously maximize the average mutation level (M) of the library and minimize the SCHEMA energy (E) of the library. Each point in the graph represents a library of chimeras, and the library that was selected is shown in black. Libraries with randomized breakpoints are shown in light gray for comparison. Source data are provided as a Source Data file.


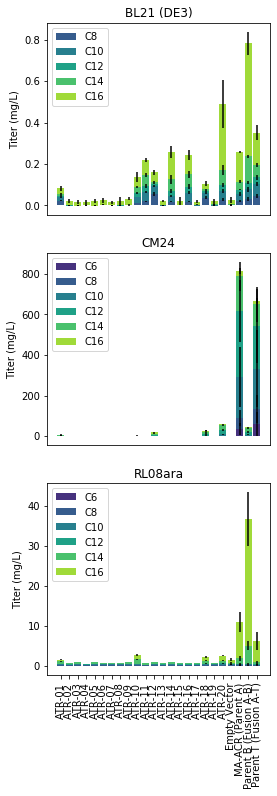


**Supplementary Figure 2**. **Total fatty alcohol titer data in BL21(DE3) (aerobic), CM24 (anaerobic) and RL08ara (aerobic) for the initial chimera seed sample.** Different carbon chain lengths (i.e. C6, C8 etc.) are shown as different colors. The total titers from BL21 (DE3) and CM24 were used to train the models used for the first round of UCB optimization, and the RL08ara data was used for all subsequent rounds. Data are presented as the mean ± standard deviation (SD) for each fatty alcohol chain length. Source data are provided as a Source Data file.


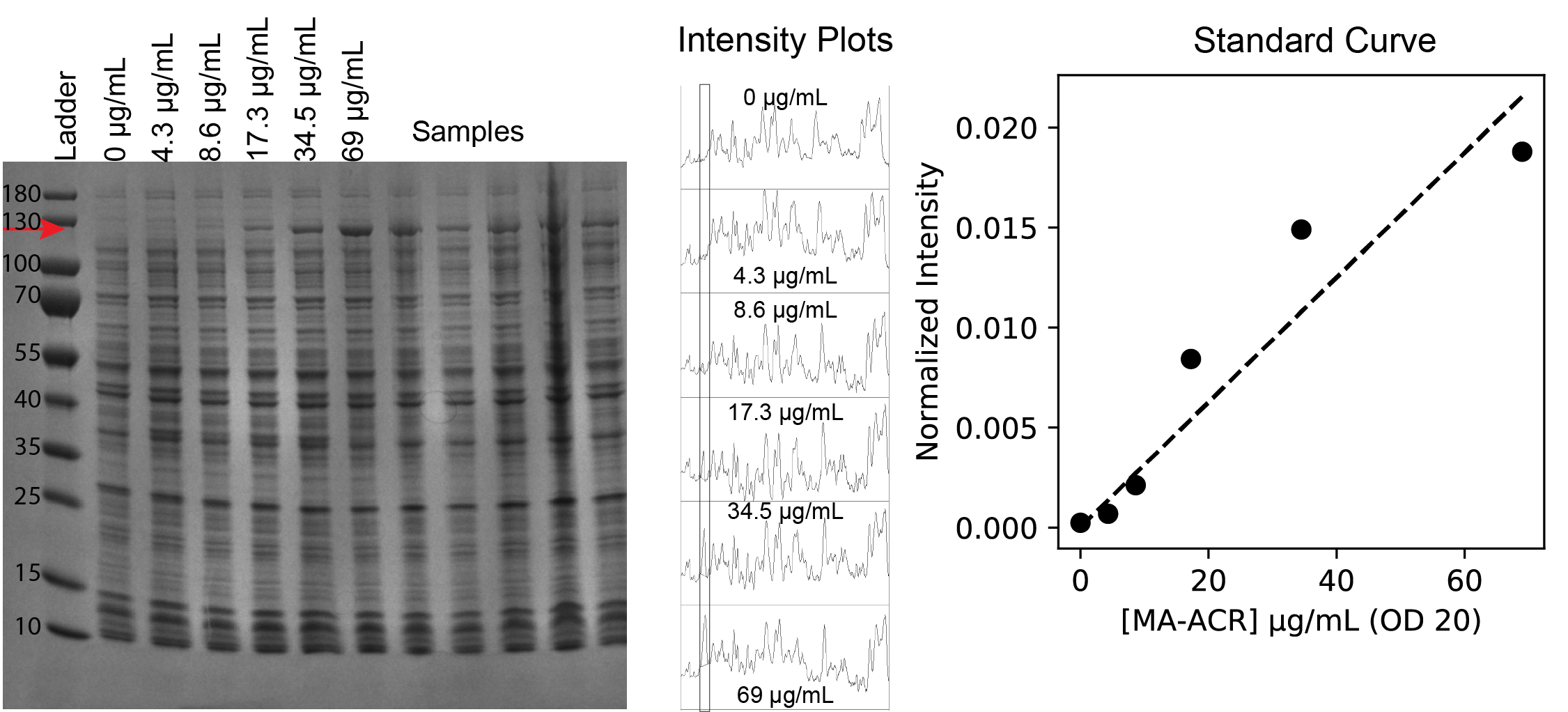


**Supplementary Figure 3**. **Representative SDS-PAGE gel for measuring FAR expression level (the molecular weight markers are in units of kDa), image analysis of the intensity plots, and standard curve.** The red arrow in the gel panel shows the expected molecular weight of MA-ACR. Source data underlying Supplementary Figure 3 gel (left) and standard curve (right) are provided as a Source Data file.


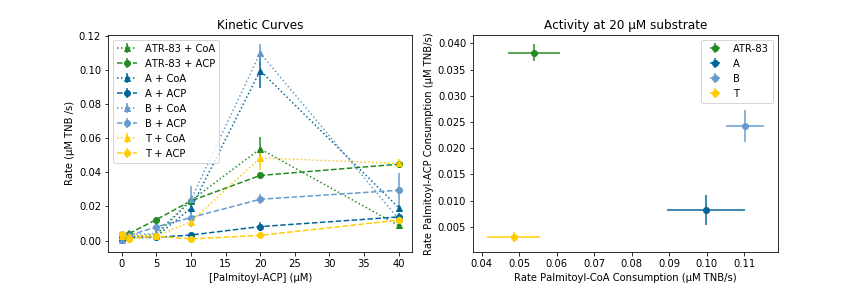


**Supplementary Figure 4**. **Comparison of enzyme activity on palmitoyl-CoA substrates and acyl-ACP substrates for the three parental enzymes (Parents A, B, and T) and ATR-83.** Data are presented as the mean of four technical replicates ± SD. Source data are provided as a Source Data file.


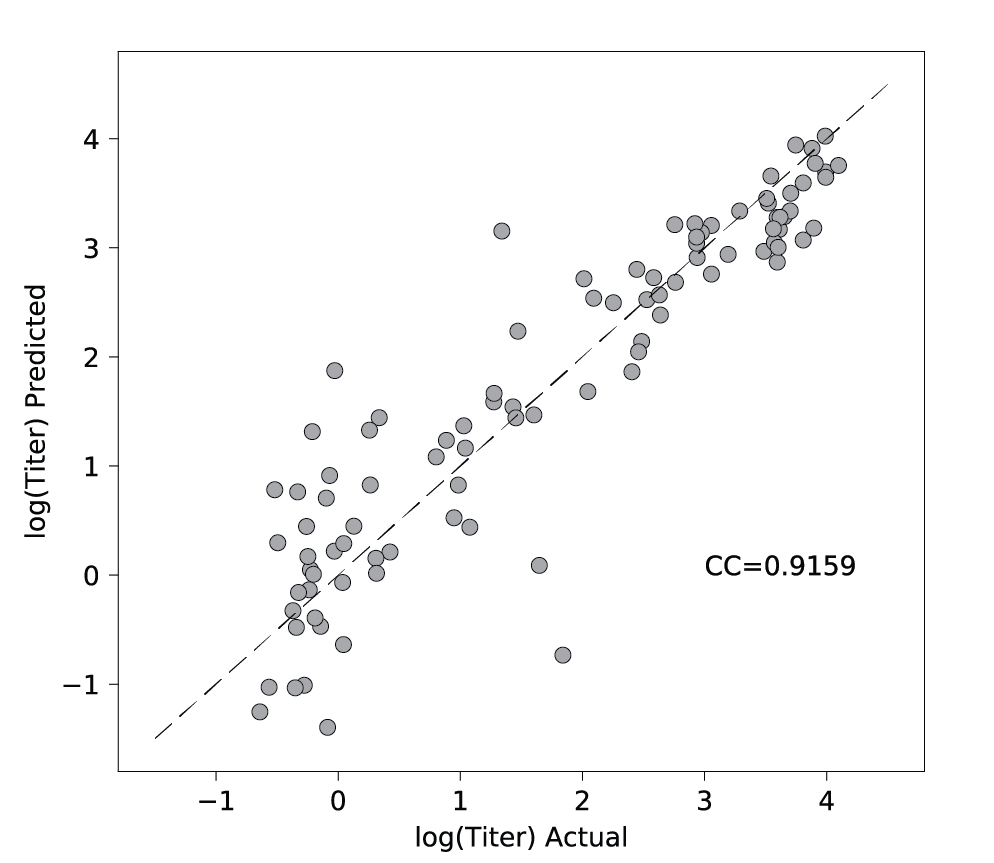


**Supplementary Figure 5**. **Cross-validated Gaussian process regression model used to study chimera landscape and determine contributions of blocks.** Source data are provided as a Source Data file.


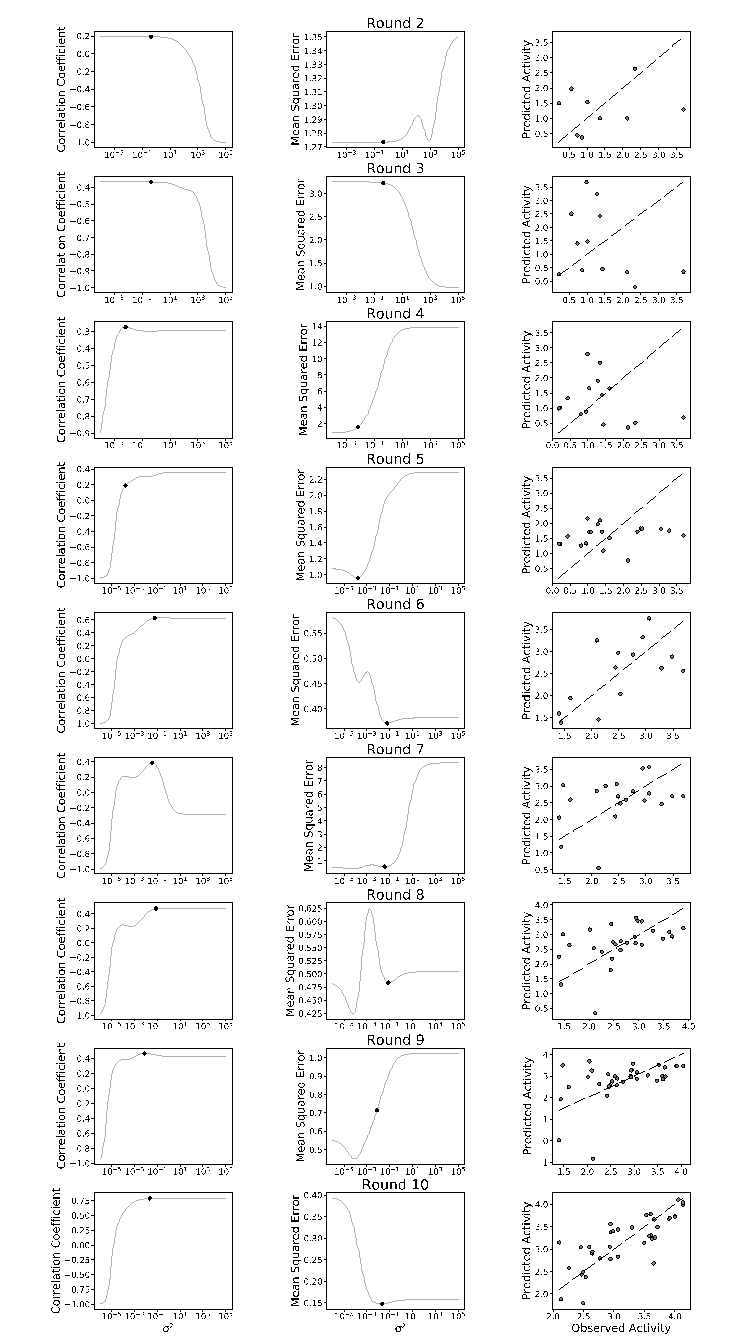


**Supplementary Figure 6. Cross validation scans by round during sequence optimization.** The leftmost column shows the correlation coefficients as a function of $\sigma^{2}$, the middle shows the mean squared error, and the right shows the final cross validated result. The selected values of $\sigma^{2}$ are shown as black dots in the leftmost and middle columns.

**Supplementary references**

(1) Rasmussen, C. E., Williams, C. *Gaussian Processes for Machine Learning*; Adaptive Computation and Machine Learning; MIT Press: Cambridge, MA (2006).

(2) Lennen, R. M., Braden, D. J., West, R. M., Dumesic, J. A., Pfleger, B. F. A process for microbial hydrocarbon synthesis: Overproduction of fatty acids in *Escherichia coli* and catalytic conversion to alkanes. *Biotechnol. Bioeng.* **106**, 193–202 (2010).

(3) Mehrer, C. R., Incha, M. R., Politz, M. C., Pfleger, B. F. Anaerobic production of medium-chain fatty alcohols via a β-reduction pathway. *Metab. Eng.* **48**, 63–71 (2018).

(4) Youngquist, J. T., Schumacher, M. H., Rose, J. P., Raines, T. C., Politz, M. C., Copeland, M. F., Pfleger, B. F. Production of medium chain length fatty alcohols from glucose in Escherichia coli. *Metab. Eng.* **20**, 177–186 (2013).

(5) Néstor, N., Hernández, J., Lozada, H., Lai, R.-Y., Simmons, T. R., Thomas, K. A., Chowdhury, R., Maranas, C. D., Pfleger, B. F. Highly active C_8_-acyl-ACP thioesterase variant isolated by a synthetic selection strategy. *ACS Synth. Biol.* **7**, 2205–2215 (2018).
