# Supplementary material for: Machine learning-guided acyl-ACP reductase engineering for improved in vivo fatty alcohol production
Source: Nat Commun. 2021 Oct 5;12:5825. doi: 10.1038/s41467-021-25831-w (PMC8492656; doi:10.1038/s41467-021-25831-w)
Supplement: Supplementary file 2 — Reporting Summary [file 41467_2021_25831_MOESM2_ESM.pdf]

Corresponding author(s): Philip Romero
Last updated by author(s): Aug 31, 2021

Reporting Summary

Nature Portfolio wishes to improve the reproducibility of the work that we publish. This form provides structure for consistency and transparency in reporting. For further information on Nature Portfolio policies, see our Editorial Policies and the Editorial Policy Checklist.

Statistics

For all statistical analyses, confirm that the following items are present in the figure legend, table legend, main text, or Methods section.

n/a	Confirmed	
☐	☒	The exact sample size (*n*) for each experimental group/condition, given as a discrete number and unit of measurement
☐	☒	A statement on whether measurements were taken from distinct samples or whether the same sample was measured repeatedly
☐	☒	The statistical test(s) used AND whether they are one- or two-sided<br>*Only common tests should be described solely by name; describe more complex techniques in the Methods section.*
☐	☒	A description of all covariates tested
☒	☐	A description of any assumptions or corrections, such as tests of normality and adjustment for multiple comparisons
☐	☒	A full description of the statistical parameters including central tendency (e.g. means) or other basic estimates (e.g. regression coefficient) AND variation (e.g. standard deviation) or associated estimates of uncertainty (e.g. confidence intervals)
☐	☒	For null hypothesis testing, the test statistic (e.g. *F*, *t*, *r*) with confidence intervals, effect sizes, degrees of freedom and *P* value noted<br>*Give P values as exact values whenever suitable.*
☐	☒	For Bayesian analysis, information on the choice of priors and Markov chain Monte Carlo settings
☒	☐	For hierarchical and complex designs, identification of the appropriate level for tests and full reporting of outcomes
☒	☐	Estimates of effect sizes (e.g. Cohen's *d*, Pearson's *r*), indicating how they were calculated

Our web collection on statistics for biologists contains articles on many of the points above.

Software and code

Policy information about availability of computer code

Data collection	GC Solution Software (Shimadzu) was used in the collection of raw GC traces<br>SparkControl (V2.3) was used to collect plate reader data
Data analysis	MODELLER (version 9) was used to make homology models used.<br>Custom python 3 scripts were used to integrate peaks from the GC traces and calculate the fatty alcohol titers. Custom python 3 scripts were also used to analyze the data and employ machine learning algorithms to aid in designing new sequences. These scripts are available at the following GitHub repository: https://github.com/RomeroLab/ML-Guided-Acyl-ACP-Reductase-Engineering<br>FIJI (version 2.1.0/1.53c) was used to analyze SDS-PAGE gels and quantify expression levels.<br>RosettaDock (version 4.0) was used to simulate ACP binding to MA-ACR.<br>PyMOL (version 2.4.2) was used to aid in structural analysis.

For manuscripts utilizing custom algorithms or software that are central to the research but not yet described in published literature, software must be made available to editors and reviewers. We strongly encourage code deposition in a community repository (e.g. GitHub). See the Nature Portfolio guidelines for submitting code & software for further information.

Data

Policy information about availability of data

All manuscripts must include a data availability statement. This statement should provide the following information, where applicable:

- Accession codes, unique identifiers, or web links for publicly available datasets
- A description of any restrictions on data availability
- For clinical datasets or third party data, please ensure that the statement adheres to our policy

The authors affirm that all data supporting the conclusions of this work are located within the paper and it's supplementary files. Data are also available on the ProtaBank server at https://www.protabank.org/study_analysis/nu9KXbjT4/
Protein structure data for the from the Protein Data Bank (PDB) was also used in this paper for the following PDB IDs: 6DFL, 3M1A, 3RKR, 3RIH, 3AFM, 3AFM, 4BMV.

Field-specific reporting

Please select the one below that is the best fit for your research. If you are not sure, read the appropriate sections before making your selection.

☒ Life sciences ☐ Behavioural & social sciences ☐ Ecological, evolutionary & environmental sciences

For a reference copy of the document with all sections, see nature.com/documents/nr-reporting-summary-flat.pdf

Life sciences study design

All studies must disclose on these points even when the disclosure is negative.

Sample size	No sample size calculations were performed. Sample sizes in each design round were limited by the number of sequences in the round and by resources such as incubator space. Typically, samples were tested in duplicate or triplicate in each round (for in vivo assays replicates were usually cultures derived from separate, individual colonies).
Data exclusions	Exclusion criteria were not pre-established. Data from one experiment characterizing AHR-ATR fusions was excluded because nearly all the titers were the same as those from the culture containing the empty vector control, including cultures expressing some sequences whose titer was already known to be high. The sequences in this experiment were subsequently re-tested multiple times. Two individual measurements of chimeric sequences were also excluded from the final analysis. One was excluded during the sequence optimization algorithm, and the other was excluded after all data had been collected. Both measurements were excluded based on extreme deviation from other replicates of the same samples and outlier analysis.
Replication	Additional biological replicates of key sequences were performed after concluding the sequence optimization. Replication was successful and the final reported means reflect the aggregate of all replicates. The number of times individual sequences were assayed in independent experiments was highly variable (more important sequences were assayed more often), but the top performing sequence was assayed in vivo in six independent experiments (for a total of 11 replicates), and the original starting sequence, MA-ACR, was assayed in vivo in 18 individual experiments (for a total of 26 replicates).
Randomization	In collection of GC data the order of the samples was randomized.
Blinding	Blinding was not relevant to this study. Our approach is an exploratory active-learning style search through a protein fitness landscape. In order to train ML models, it was necessary to know the mapping of the proteins sequence to it's function. Models were evaluated using leave-one-out cross validation, and each optimization round models were applied to predict activities of sequences not yet observed by the model. This information was used to design new sequences, and the activity level of these new sequences was not truly known until after they were tested.

Reporting for specific materials, systems and methods

We require information from authors about some types of materials, experimental systems and methods used in many studies. Here, indicate whether each material, system or method listed is relevant to your study. If you are not sure if a list item applies to your research, read the appropriate section before selecting a response.

Materials & experimental systems

n/a	Involved in the study
☒	☐ Antibodies
☒	☐ Eukaryotic cell lines
☒	☐ Palaeontology and archaeology
☒	☐ Animals and other organisms
☒	☐ Human research participants
☒	☐ Clinical data
☒	☐ Dual use research of concern

Methods

n/a	Involved in the study
☒	☐ ChIP-seq
☒	☐ Flow cytometry
☒	☐ MRI-based neuroimaging
